# The Molecular Biology Toolkit (MBT): a modular platform for developing molecular visualization applications

**DOI:** 10.1186/1471-2105-6-21

**Published:** 2005-02-06

**Authors:** John L Moreland, Apostol Gramada, Oleksandr V Buzko, Qing Zhang, Philip E Bourne

**Affiliations:** 1 San Diego Supercomputer Center, 9500 Gilman Drive, La Jolla, CA 92093, USA; 2 Department of Pharmacology, University of California San Diego, 9500 Gilman Drive, La Jolla, CA 92093, USA

## Abstract

**Background:**

The large amount of data that are currently produced in the biological sciences can no longer be explored and visualized efficiently with traditional, specialized software. Instead, new capabilities are needed that offer flexibility, rapid application development and deployment as standalone applications or available through the Web.

**Results:**

We describe a new software toolkit – the Molecular Biology Toolkit (MBT; ) – that enables fast development of applications for protein analysis and visualization. The toolkit is written in Java, thus offering platform-independence and Internet delivery capabilities. Several applications of the toolkit are introduced to illustrate the functionality that can be achieved.

**Conclusions:**

The MBT provides a well-organized assortment of core classes that provide a uniform data model for the description of biological structures and automate most common tasks associated with the development of applications in the molecular sciences (data loading, derivation of typical structural information, visualization of sequence and standard structural entities).

## Background

Recent scientific and technical advances in the field of experimental biology, particularly in genomics, have produced large amounts of biological data, which has posed new conceptual challenges. The visualization and visualization-driven analysis of these experimentally derived data has become a key component of the scientific process.

Until recently, these needs were typically approached by designing applications specialized in a set of well-defined specific tasks. So, for example, popular applications for molecular visualization include, but are not limited to, Molscript [1], PyMol [2], Rasmol [3] and Swiss-PdbViewer [4]. However, the analysis of these molecular data frequently requires novel approaches to visualization and integration with a variety of data types. Therefore, the ability to quickly prototype and develop software suitable for diverse tasks becomes paramount. Hence, libraries, like the MBT described here, are particularly useful, especially given that many applications can be accessed through the Web.

This paper, aimed at bioinformatics software developers, provides a concise presentation of the design and capabilities of the toolkit, presents a number of models for its usage, and illustrates its performance with several applications. The paper is organized as follows. The following section provides a general overview of the application programming interface (API). The Results and Discussion section presents details about core components of the toolkit and illustrates its capabilities with two applications. The Conclusions section summarizes the main functionality of the toolkit, discusses availability and documentation, presents performance data and outlines future development plans.

## Implementation

The application-programming interface (API) that serves as the foundation of the toolkit has emerged from the exploration of the typical needs encountered by a researcher in the analysis of a documented biological molecule. The list of requirements includes reading the data file and converting the information to data objects that are easily accessible by applications, extracting subsets of chemical components according to different chemical or biological properties of interest (e.g. chains, residues, CA atoms, ligands, etc.), deriving information that is not explicitly present in the original input data (e.g., covalent and hydrogen bonds, torsion angles, secondary structures) and visualizing chemical or physical properties of different subsets of the molecule, or the entire molecule.

**Figure 1:**
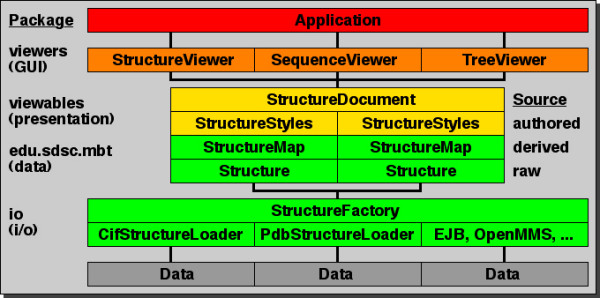
The architecture of the toolkit.

Based on the requirements identified above, we have arrived at the multi-layered design illustrated in Fig. 1. The bottom (input/output) layer contains facilities for importing molecular data from a variety of sources. The *StructureFactory *class offers a uniform approach to loading structural data, independent of the format of the source. The class makes use of a set of loaders that can import data from a variety of sources, either on the same machine or located on a remote server. Moreover, this allows the developer to write applications that do not have to uniquely specify the source of data. Instead, a number of methods in the *StructureFactory *class enable loading of structures based on a series of source descriptors: file name, PDB id code, URL location, etc. The first loader capable of handling the source descriptor is used thus providing a data access and retrieval mechanism that is transparent to the user.

The output of the load methods in the *StructureFactory *is a *Structure *object, which is effectively an interface to a summary of raw primary and secondary structure data. The *Structure *object is not restricted to holding structural information and can contain any other data relevant to the organization of the molecular entity. For instance, it can store the description of single or multiple protein sequences preserving the ordering and alignment of the amino acid residues and their properties.

The *StructureMap *class builds the internal data model of the simple or complex molecule and provides hierarchical access to both raw data and derived information generated from the input. This includes access to chains, residues, atoms, bonds, nucleic acid components, ligand atoms, fragments associated with secondary structure components, or features defined by the user.

The *StructureStyles *class provides information about rendering, coloring and selection attributes associated with any structure component produced by the *StructureMap*. A wide variety of methods in this class allow any module of an application, in particular any viewer, to set and retrieve the information describing the visually represented parameters of any structure component.

The *StructureDocument *is designed as a container class that maintains a log of all loaded structures and viewers that are instantiated by an application in a given session. It also has the role of generating events associated with the addition or removal of structures and viewers.

The next level of the API contains graphical user interface (GUI) elements. This portion of the package contains high level constructs, such as windows and panels designed to display 3D graphics, protein or DNA sequences or tree representations of the chemical components of the molecule, as well as lower level components that can be used to build a molecular scene according to a developer's preferences.

Finally, at the application level, a number of applications are provided that can be used to illustrate the features of the toolkit, or as starting templates for new application development. For example, the applications illustrate how to use methods in the *StructureMap *class to retrieve all secondary structures within a given molecule, or how to obtain a list of atoms or residues. This is the level that most developers would modify in order to create custom applications tailored to their specific needs.

Note that care has been taken to further enable application developers to use different components of the toolkit to build purely analytical applications that have no visualization component. For example, a developer could write a command-line tool that simply loads a molecule and gathers statistics about the structure data without presenting any graphical interface. Such an application could be part of a back-end process that runs in a web-server environment.

## Results and discussion

### Core components

The MBT was developed as an object-oriented Java-based environment and hence is flexible, modular and lightweight, which facilitates maintenance, web deliverability and limits the required computer resources. Moreover, the toolkit can be easily extended and having been written completely in Java is effectively platform-independent.

**Figure 2:**
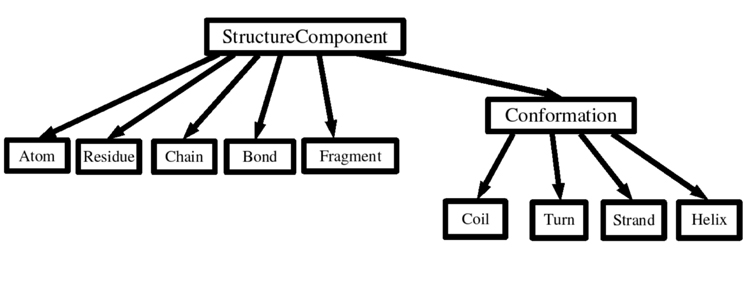
The MBT data model.

The internal data model describes the hierarchical organization of a protein molecule – Structure, Chain, Residue, and Atom. We have implemented this data model by designing a class hierarchy to efficiently encode its elements. The components of this class hierarchy (Fig. 2) are built around a *Structure *object (Fig. 1). Structure is a container designed to hold all raw information pertaining to the given unit of biological information: protein sequence, genomic sequence, taxonomy information, experimental data and so on. The remainder of the class hierarchy represents the components of the macromolecule. The toolkit is not inherently limited to operating on biological molecules, and can easily be used to manipulate small organic and inorganic molecules. Selection, rendering and coloring attributes for all biological molecules are handled by *StructureStyles*.

The *Fragment *class contains, for example, one of the four known conformations: α-helix, β-strand, turn or random coil, but is sufficiently general to define ranges of residues grouped according to any property of interest. In addition, the data model contains classes that describe other objects associated with derived information, such as covalent or hydrogen bonds.

In order to provide uniform style features across the toolkit a *StructureStyles *class has been implemented. This class maintains a representation of the rendering characteristics of all structure component objects so that any application module has access to the style data for any given object that needs to be visually represented. The set of style parameters maintained by the *StructureStyles *class is comprehensive – it is the union of the sets of style parameters required by any known viewer.

Communication between different components of the toolkit is enabled by a flexible event handling mechanism. Changes in the data, rendering styles, addition or removal of viewers and many other actions with toolkit-wide impact generate descriptive events, which are recursively propagated across the toolkit components, allowing an automatic synchronization of the state of different active parts of an application.

Version 1.0 of the MBT provides three standard viewers: a 3D structure viewer, a primary sequence viewer, and a tree viewer. The 3D viewer is implemented using the Java3D™ extension. The use of Java3D for visualization was motivated by the convenience of the availability of high-level constructs for building complex 3D scenes. Analysis of the performance aspects [5] of Java3D has shown that some performance issues can be overcome through a careful organization of the molecular scene. Existing applications indicate that the visualization of most molecules using typical desktops and graphics cards is fast and fully interactive. For example, typical protein data sets with four to five thousand atoms (e.g., PDB identifiers 4 HHB, 10 MH, 6 GEP) load and display in four or five seconds on a Pentium III 1.2 GHz laptop computer.

**Figure 3:**
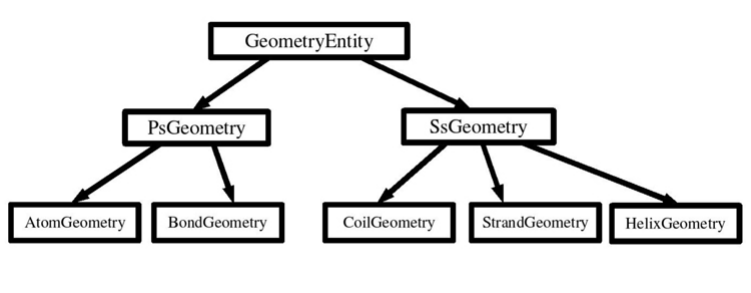
The class hierarchy of the geometry engine.

A schematic representation of the data structure used by the 3D viewer is shown in Figure 3. For each primary or secondary structure element, a geometry object (*GeometryEntity*) is built, which is then attached as a *BranchGroup *node to a *SceneGraphObject *(the common superclass for all graph objects in Java3D) representing the three-dimensional image of the molecule. The *PsGeometry *(primary structure) and *SsGeometry *(secondary structure) classes provide a number of methods that build complete 3D scenes from a given set of primary or secondary structure data object (See Fig. 2).

The geometry engine of the 3D viewer uses a flexible approach to generate ribbon-like surfaces. It allows the construction of ribbons using an extrusion with any shape of the cross section. A few most commonly used shapes are immediately available as core components of the toolkit. However, the developers could easily implement and register with the toolkit any additional shapes that may be of interest in their specific applications. The quality of geometry can be controlled either directly by setting individual geometry rendering parameters, or indirectly by a general quality parameter that optimizes the number of facets/vertices used in the construction of different geometric shapes. This allows for an easy adjustment of the application parameters in a wide performance-quality range, from very fast line-only drawing, to a somewhat slower, publication-quality rendering.

The sequence viewer is a module designed to display primary sequences of proteins and nucleic acids that are either derived from the loaded structures or acquired from individual sequence files or sequence alignments. The sequence viewer uses AWT drawing methods, which does not impose any specific requirements on the client system, as they are part of the standard Java distribution. The viewer is designed as a full-featured module capable of performing most of the sequence analysis tasks, including basic statistics, pattern and motif searching and display of secondary structure mapping onto the sequence. The viewer is capable of displaying an unlimited number of sequences and provides multiple representation options. The latter include residue coloring by several criteria with a possibility of an easy extension, setting sequence display to any of the available system fonts, a flexible selection system, and more. As stated, the integrated event handling of the toolkit allows for simultaneous updates of the presentation layer for any participating viewers. Hence, the toolkit has the built-in support for common selection and common coloring across all registered viewers. This offers an important visual cue to many applications, linking for example sequence and structural components.

The tree viewer offers a hierarchical view of all components of a given molecule. It reflects the logical organization of the derived *StructureMap *data including *Molecule*, *Chain*, *Residue*, and *Atom *objects. The tree view provides a convenient mechanism to select portions of a molecule based upon the biological relationship between atoms, residues, and chains.

Finally, the MBT provides a repository of data and methods that can be used for the retrieval and/or derivation of physical, chemical and structural information associated with the molecules loaded by an application. For example, the package contains a periodic table with physico-chemical properties of the elements, as well as methods for the derivation of the secondary structure information, using the Kabsch-Sander algorithm [6]. Full details of all these features are provided with the documentation.

### Applications built using the MBT

Applications can be explored and downloaded from the MBT Website [7]. They have been tested on a variety of UNIX, Windows and MAC OS X platforms.

**Figure 4:**
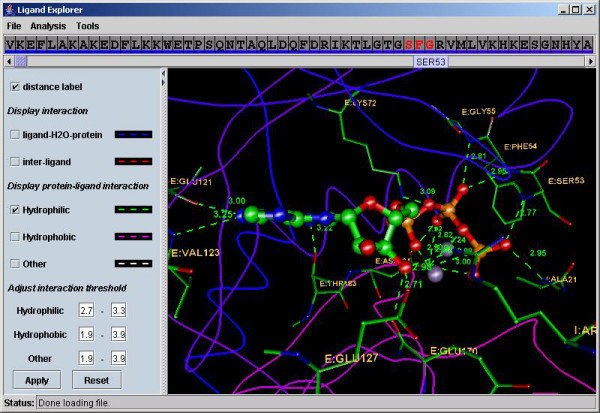
Ligand Explorer. The structure of cAMP dependant protein kinase (PDB id 1ATP) showing the hydrophilic interactions of the bound ATP to the protein and bound inhibitor which mimics substrate in the phosphotransfer reaction from the gamma phosphate of ATP.

The Ligand Explorer [8] (a.k.a. LigPro; Figure 4) is an integral part of the reengineered RCSB Protein Data Bank (PDB) [9,10], which is currently in beta testing. In the present PDB, a user interested in protein-ligand interactions must download the structure, decide on a graphics program and likely learn a scripting language to provide details of hydrophilic and hydrophobic interactions between protein and ligand at different cutoff distances. Ligand Explorer achieves this at the push of a button. This produces a view with all ligands highlighted. The user then selects a ligand for a review of detailed interactions. Ligand Explorer can be downloaded as a separate application and used to access local files or files on the PDB's servers.

**Figure 5:**
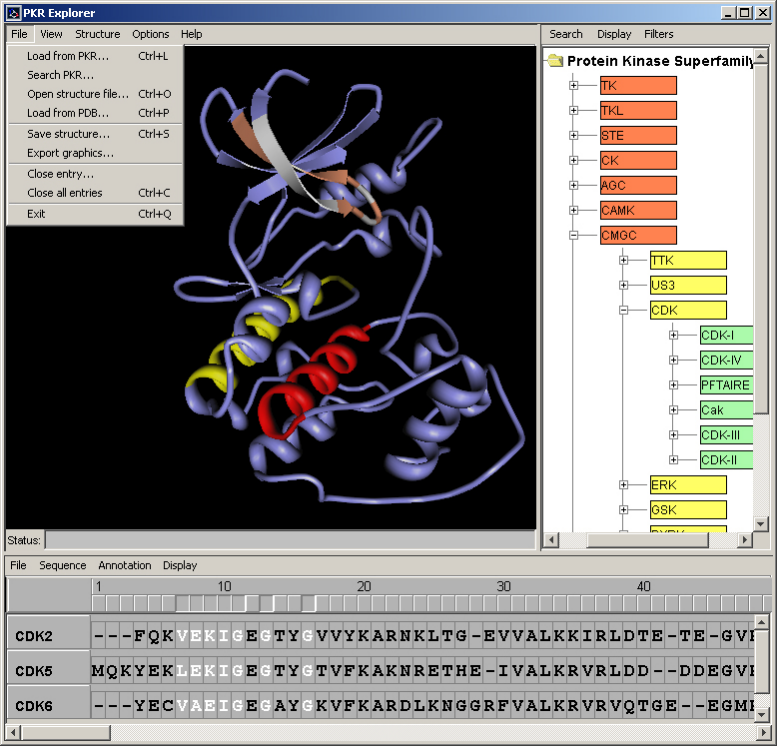
Protein kinase explorer. An alternative view of a cAMP dependant protein kinase with residues from the multiple structure alignment mapped to the corresponding aligned sequences and to a single template structure from the set.

The protein kinase exploration tool (Fig 5.) is part of the new protein kinase resource [11]. It uses the 3D viewer supplied by the toolkit with a few modifications that allow more extensive coloring and rendering options. The multiple sequence viewer presents the multiple sequence alignments resulting from the multiple structure alignments which are stored in the database. Another viewer displays the superfamily relationship of the sequences present in the database.

## Conclusions

The molecular biology toolkit (MBT) provides a set of pluggable and extensible classes for use by application developers interested in the visualization and analysis of macromolecular data. The MBT provides a set of pre-written data loaders, viewers, a common data model and the means to add to and customize the toolkit for specific applications delivered as applets through the Web or as standalone applications. Base functionality and comparable tools (where applicable) are as follows:

• Classes to load raw data from a number of common protein structure and sequence data sources (PDB, mmCIF, FASTA, etc.) and a means to easily add new "loader" modules independent of the applications they might serve.

• A common data model to which raw information is imported, mapped, and indexed. A number of data record types (*StructureComponent *objects) are provided (e.g., Atom, Bond, Residue, Chain) and new data types are easily registered. Further the data model provides an extensible means to describe viewable or visible attributes (e.g., color, radius, drawing style) of these objects.

• Written entirely in Java, programs may be embedded (using Java WebStart or the Java Plug-In) directly inside web pages. This enables the deployment of tightly coupled interactive web content much like the popular MDL Chime plug-in.

• Applications are not restricted to the features provided by the MBT APIs. The Programmers Guide details how to extend the system.

• With source code provided, core features of the toolkit may be directly modified or extended for independent use (though, this may cause your code to diverge from and become incompatible with subsequent releases of MBT). However, adding code within the existing framework and contributing it back for others to use is encouraged. The source code has been extensively commented to produce a rich and complete set of hyper-linked javadoc API documents.

• A set of pre-written viewers (Sequence, Structure, Tree) that can be extended, replaced, omitted, or augmented with completely new viewers that implement entirely new visualization techniques.

• The 3D *StructureViewer *module provides visual representations similar to RasMol and Molscript such as balls-and-sticks, CPK spheres, split-bonds, extrusion/ribbon-style backbone traces, and secondary structure cartoons (Helix, Turn, Coil, Strand). It also provides 3D labeling.

• A scripting interface is being developed for MBT to enable toolkit functionality to be command-line or even script-driven (similar to MDL Chime or PyMol).

Version 1.0 of the toolkit and sample applications, including those described here, are available for download from the project web page [7]. The same site contains links to various documentation pages (Project Introduction, Talk/Presentation Slides, Related Links, Installation Guide, Build Guide, Programmers Guide, Examples Source, and Toolkit API). The MBT has been tested on common hardware and UNIX, Windows and MAC OS X operating systems. A good consumer-level graphics card is recommended. The loading and generation of a 3D scene when representing a typical protein structure takes a few seconds. Large structures from the PDB [9] that contain over 10^5 ^atoms require physical memory in excess of 500 MBytes and on a notebook computer with a 1.2 GHz processor can take nearly one minute. Efforts at optimization are on-going.

New applications are on-going including the generation of high quality images for all structures in the Protein Data Bank and new ways of visualizing protein-protein interactions. We invite contributions to the MBT by sending mail to mbt@sdsc.edu. Bugs may be reported to a bug tracker available on the project web site [7].

## Availability and requirements

• **Project Name: **Molecular Biology Toolkit (MBT)

• **Project Home Page: **

• **Operating System: **Platform independent

• **Programming Language: **Java

• **Other requirements: **Java 1.3.1 or higher, Java3D

• **License: **Free for educational, research and non-profit purposes

• **Any restrictions to use by non-academics: **Contact the University of California at San Diego's Technology Transfer Office (invent@ucsd.edu, 1-858-534-5815)

## Authors' contributions

JLM is one of the designers of the API and co-developer of the toolkit. AG designed and implemented the geometry generation modules, implemented the algorithms for secondary structure generation and bond detection, and drafted the paper. OVB developed the PKR Explorer. QZ developed the Ligand Explorer. PEB coordinated the whole project, suggesting the general functionality and scientific objectives of the toolkit.
